# Supplementary material for: In Vivo PET Imaging of Monocytes Labeled with [89Zr]Zr-PLGA-NH2 Nanoparticles in Tumor and Staphylococcus aureus Infection Models
Source: Cancers (Basel). 2021 Oct 10;13(20):5069. doi: 10.3390/cancers13205069 (PMC8533969; doi:10.3390/cancers13205069)
Supplement: Supplementary file 1 [file cancers-13-05069-s001.zip › cancers-1380533 Supplementary.pdf]

cancers

MDPI

Supplementary Materials: In Vivo PET Imaging of Monocytes Labeled with [89Zr]Zr-PLGA-NH2 Nanoparticles in Tumor and *Staphylococcus aureus* Infection Models

Massis Krekorian, Kimberley R.G. Cortenbach, Milou Boswinkel, Annemarie Kip, Gerben M. Franssen, Andor Veltien, Tom W.J. Scheenen, René Raavé, N. Koen van Riessen, Mangala Srinivas, I. Jolanda M. de Vries, Carl G. Figdor, Erik H.J.G. Aarntzen and Sandra Heskamp

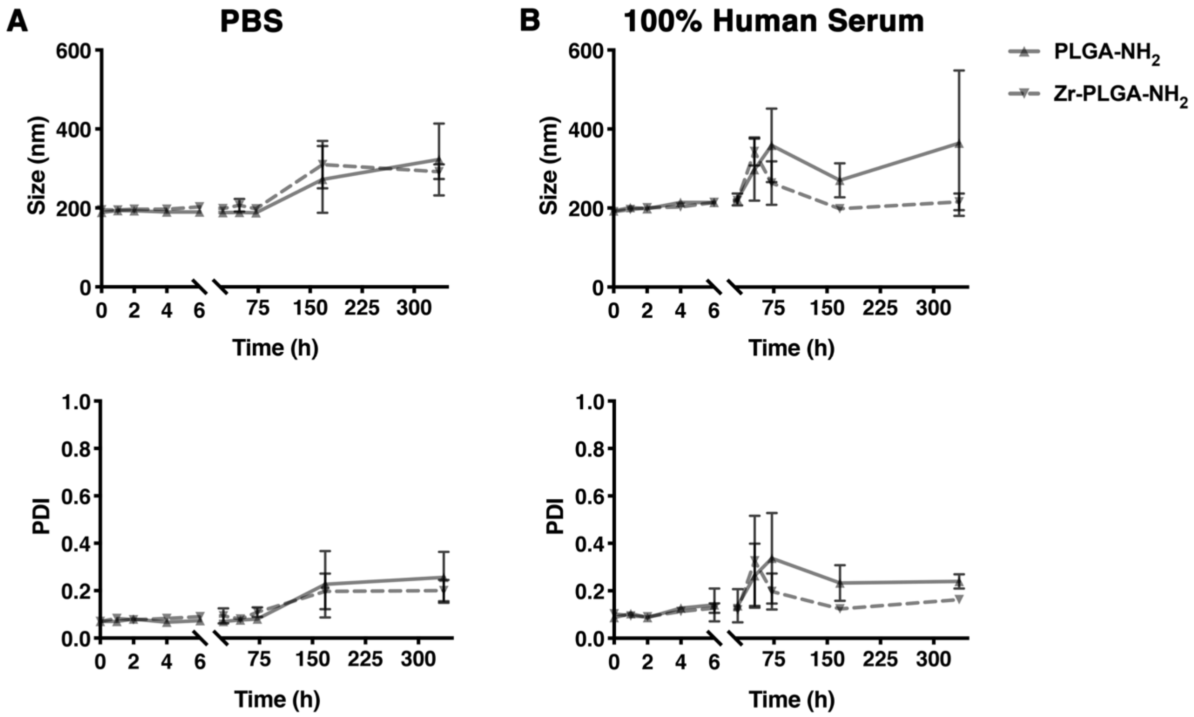

Figure S1. Over time particle stability in different buffers. Diameter and PDI measurements of PLGA-NH2 and Zr-PLGA-NH2 NPs incubated in (**A**) PBS and (**B**) 100% human serum at 37 °C for 0, 1, 2, 4, 6, 24, 48, 72, 168 and 336 hours. The mean and standard deviation of at least three independent experimental data sets is shown. Abbreviations: Poly(lactic-co-glycolic acid) with primary amine nanoparticles (PLGA-NH2 NPs) and non-radioactive zirconium-labelled PLGA-NH2 NPs (Zr-PLGA-NH2).

Table S1. Biodistribution of [89Zr]Zr-PLGA-NH2 NPs at day 3 and 14 after intravenous tail injection in C57BL/6 mice. Data is expressed as %Injected Dose per gram (mean ± standard deviation, *n* = 3).

Organs	Day 3	Day 14
Blood	0.40 ± 0.46	0.16 ± 0.09
Spleen	0.23 ± 0.07 × 10^3	0.18 ± 0.03 × 10^3
Liver	27.47 ± 3.38	17.00 ± 1.85
Kidney	0.51 ± 0.09	0.48 ± 0.06
Heart	0.50 ± 0.04	0.54 ± 0.13
Lung	2.39 ± 0.60	3.02 ± 1.15
Pancreas	0.29 ± 0.04	0.23 ± 0.06
Bladder	0.35 ± 0.03	0.51 ± 0.08
Duodenum	0.70 ± 0.16	0.26 ± 0.08
Ileum	0.78 ± 0.38	0.36 ± 0.11
Colon	0.15 ± 0.02	0.29 ± 0.21
Brain	0.02 ± 0.00	0.04 ± 0.02

Muscle	0.32 ± 0.11	0.16 ± 0.05
LN (inguinal)*	1.34 ± 0.23	2.42 ± 0.84
Femur	6.07 ± 2.64	5.89 ± 0.06
Bone marrow	14.90 ± 5.81	14.08 ± 2.70
Thymus	0.33 ± 0.08	0.25 ± 0.04
Brown fat	0.94 ± 0.19	1.07 ± 0.25
Stomach	0.32 ± 0.02	0.24 ± 0.04
Salivary gland	0.43 ± 0.11	0.36 ± 0.07
Knee	8.67 ± 2.20	7.24 ± 1.77

* Abbreviations: LN, lymph node.

Table S2. Biodistribution of [^{89}Zr]Zr-THP-1 cells at 24 hours after subcutaneous injection. Data is expressed as %Injected Dose per gram (mean ± standard deviation, *n* = 4).

Organs	[^{89}Zr]Zr-PLGA-NH_2 NPs	10,000 [^{89}Zr]Zr-THP-1 cells	100,000 [^{89}Zr]Zr-THP-1 cells
Blood	1.22 ± 0.43	0.81 ± 0.79	0.59 ± 0.11
Matrigel	2.21 ± 1.19 × 10^3	0.62 ± 0.44 × 10^3	1.00 ± 0.25 x 10^3
Salivary gland	0.58 ± 0.14	0.44 ± 0.49	0.29 ± 0.12
LN (inguinal)*	2.54 ± 1.84	15.24 ± 12.74	2.93 ± 2.33
Heart	0.41 ± 0.08	0.58 ± 0.56	0.20 ± 0.05
Lung	1.27 ± 0.17	0.60 ± 0.41	0.44 ± 0.16
Thymus	1.04 ± 0.23	1.76 ± 2.48	0.57 ± 0.50
Bladder	0.89 ± 0.35	4.77 ± 4.10	1.10 ± 0.87
Liver	0.74 ± 0.23	0.83 ± 0.47	0.25 ± 0.02
Stomach	0.33 ± 0.06	1.01 ± 0.51	0.15 ± 0.04
Duodenum	0.45 ± 0.20	1.09 ± 0.68	0.24 ± 0.06
Ileum	0.69 ± 0.23	1.74 ± 0.60	0.31 ± 0.07
Colon	0.50 ± 0.34	0.75 ± 0.42	0.18 ± 0.07
Pancreas	0.40 ± 0.06	0.47 ± 0.19	0.17 ± 0.06
Spleen	0.41 ± 0.10	0.61 ± 0.42	0.19 ± 0.04
Kidney	0.77 ± 0.14	0.71 ± 0.26	0.33 ± 0.06
Muscle	0.24 ± 0.07	0.49 ± 0.41	0.10 ± 0.08
Knee	5.30 ± 1.52	2.24 ± 1.60	2.16 ± 0.45
Bone marrow	1.81 ± 0.70	5.54 ± 4.88	1.35 ± 1.55
Femur	4.15 ± 0.98	2.00 ± 1.73	1.56 ± 0.71
Brown fat	0.64 ± 0.20	0.68 ± 0.62	0.36 ± 0.05
Brain	0.07 ± 0.03	0.13 ± 0.06	0.04 ± 0.01

* Abbreviations: LN, lymph node.

Table S3. Biodistribution of [^{89}Zr]Zr-THP-1 cells at 24 hours after intravenous injection in *Staphylococcus aureus* and MDA-MB-231 tumor models. Data is expressed as %Injected Dose per gram (mean ± standard deviation, *n* = 4–5).

Organs	*Staphylococcus aureus*	MDA-MB-231 Tumor
Blood	2.14 ± 0.65	3.80 ± 4.04
Infected Muscle (R)*	2.66 ± 0.95	-
Control Muscle (L)*	0.32 ± 0.08	-
Tumor	-	1.17 ± 0.24
Muscle foreleg (R)*	0.49 ± 0.08	0.46 ± 0.19
Salivary gland	0.63 ± 0.09	0.62 ± 0.11
LN *	2.62 ± 0.41	3.49 ± 1.00
Heart	0.69 ± 0.11	0.59 ± 0.07
Lung	43.19 ± 16.84	31.44 ± 9.86
Thymus	2.04 ± 0.27	2.03 ± 0.51
Bladder	1.41 ± 0.51	1.12 ± 0.30
Liver	48.28 ± 5.88	51.37 ± 5.89
Stomach	0.50 ± 0.20	0.47 ± 0.08
Duodenum	0.87 ± 0.25	0.64 ± 0.17
Ileum	0.68 ± 0.20	0.74 ± 0.12

Colon	0.43 ± 0.11	0.40 ± 0.08
Pancreas	0.46 ± 0.13	0.46 ± 0.06
Spleen	46.67 ± 8.99	53.84 ± 20.78
Kidney	1.59 ± 0.22	2.82 ± 0.65
Knee	6.41 ± 2.40	8.82 ± 3.01
Bone Marrow	10.98 ± 3.35	21.64 ± 12.25
Femur	5.19 ± 1.65	5.99 ± 3.64
Brown Fat	1.43 ± 0.06	0.92 ± 0.15
Brain	0.09 ± 0.02	0.08 ± 0.01

* Abbreviations: LN, lymph node; Infection Muscle (R), Staphylococcus aureus in right hind leg; Control Muscle (L), PBS + Blood in left hind leg; Muscle foreleg (R), without injection.
